# Hematological changes in human lymphotropic-T virus type 1 carriers

**DOI:** 10.3389/fmicb.2022.1003047

**Published:** 2022-10-24

**Authors:** Jairo Falcão Ribeiro, Akim Felipe Santos Nobre, Louise Canto Ferreira Covre, Maria de Nazaré do Socorro de Almeida Viana, Ingrid Christiane Silva, Leonardo Miranda dos Santos, Edna Aoba Ishikawa, Carlos Araújo da Costa, Maísa Silva de Sousa

**Affiliations:** Nucleus of Tropical Medicine, Federal University of Pará, Belém, Pará, Brazil

**Keywords:** HTLV-1, hematological changes, ATL, epidemiology, Amazon

## Abstract

The human T-lymphotropic virus type 1 (HTLV-1), isolated in 1980, causes T-cell leukemia/lymphoma in adulthood, a type of lymphoproliferative disease, and chronic HTLV-1-associated myelopathy, a disease that causes paralysis of the lower limbs, which occur in about 5% of cases in this viral infection. This study aimed to establish the hematological profile of patients with HTLV-1 infection in Belém do Pará, describing the hematological parameters under study, estimating the frequency of lymphocytic atypical, and associating the hematological profile with diseases and symptoms. Hematologic data from 202 individuals were analyzed, including 87 HTLV-1 infected individuals and 115 non-HTLV-1 infected individuals as a control group, composed, at a great part, of relatives of the infected. The seroprevalence of HTLV-1 infection was observed in 71.3% of female individuals, with predominance in the group older than 50 years (44.8%). The analysis of hematological parameters showed a significant difference in the counts of the segmented cells (*p* = 0.0303) and eosinophils (*p* = 0.0092) in HTLV-1 carriers. Lymphocytic atypical was a finding present only in HTLV-1 carriers (*p* = 0.0001). There was no high frequency in the leukocyte counts of those infected by HTLV-1 not among them concerning a significant increase or decrease. It is concluded that HTLV-1 infection is prominent in women over 50 years old. The hematological profile of those infected shows a reduction of segmented cells, an increase of eosinophils, and the presence of atypical lymphocytes. The hematological profile of the HTLV-1 carrier should always be evaluated to identify early some diseases associated with the infection.

## Introduction

The human T-lymphotropic virus type 1 (HTLV-1), isolated in 1980, is a slow-progression virus, being considered of low morbidity, is related to some diseases with emphasis on adult T-cell Leukemia/Lymphoma (ATL) and chronic myelopathy, occurring in about 5% of cases of virus infection (Poiesz et al., 1980). HTLV-2 was discovered in 1982 when it was isolated from a patient with hairy cell leukemia. In Brazil, this virus has been found in indigenous populations in the Amazon, injecting drug users in urban areas, and blood donors. It is estimated that more than 2.5 million people are infected with HTLV-1 in Brazil, evidencing a concern about these data, showing that there are endemic regions regarding infection by this virus. The existence of an association between HTLV-1 infection and hematological disorders brings the importance of hematological follow-up through the performance of the blood count of infected patients, which is a tool for the evaluation of various situations, such as the aid of a diagnosis to the evolution of hematological diseases in infectious conditions.

Numerous factors can influence the hematological picture of individuals. The data provided by the blood count are essential within the investigation of hematological diseases. The diversity of information that the blood count can provide, although it is generally quite non-specific, makes this examination one of the most requested in clinical and surgical practices.

During the last decades, there has been a great technological evolution in the performance of the blood count, and manual techniques have been replaced by automated systems that present greater precision in the results and in a shorter time interval. The importance of hematology as a means of study for signs of certain diseases is very clear, helping professionals to establish possible diagnoses, establish prognoses, and monitor evolving patients.

The human T-lymphotropic virus type 1 is associated with the development of serious diseases, such as ATL and this morbidity and mortality may be higher than has been considered (Poetker et al., 2011). The morphological exam through the hematological slides of lymphoid cells is often the first sign to be awakened for the diagnosis of ATL. Lymphocytes are characterized by marked cellular pleomorphism, nuclear irregularities, and variable nuclear chromatin condensation. The most typical ATL cells are medium-sized lymphocytes with polylobulated nuclei (*flower cells*). The cytoplasm is often scarce and the nucleus is irregular (Silva et al., 2002).

Despite being a follow-up test for infected patients, few studies report the results of hematological studies of people with HTLV-1 (Catalan-Soares et al., 2001). It is important to expand the study of the hematological profile with HTLV-1 infection, due to its relationship with oncohematological diseases, in addition to enabling better clinical and laboratory follow-up and specialized counseling of individuals infected with the virus. This work aims to stabilize the hematological profile of HTLV-1 infection portals living in Belém of Pará, Brazil.

## Materials and methods

### Study site

The Center for Tropical Medicine (NTM), a unit of the Federal University of Pará (UFPA), develops different projects that benefit the population by offering free outpatient and laboratory care in the diagnosis of different tropical diseases.

The Laboratory of Molecular and Cellular Biology (LMCB) of NTM/UFPA develops teaching, research, and extension projects, aiming at the training, and improvement of its human resources. The lines of research and extension are focused on the prevention and study of sexually transmitted infections/diseases (STIs/STD), more specifically Human Papillomavirus (HPV), *Chlamydia trachomatis* (*C. trachomatis*), and HTLV.

In LMCB serological and molecular tests are performed for the detection and diagnosis of HTLV.

### Ethical aspects

The present study was submitted and approved (CAAE: 31014114.2.0000.5172) by the research ethics committee of the NMT/UFPA, in compliance with the norms and guidelines of the Federal University of Pará.

### Population, sample, and study period

This study is aimed at all people assisted by the Molecular Biology Laboratory and or the Center for Tropical Medicine of UFPA, carriers or not of the human T-lymphotropic virus, from September 2015 to August 2016. The study included 202 individuals, among them 87 people infected with HTLV-1 (Case Group) and 115 individuals negative for HTLV-1 (Control Group), mostly relatives of carriers of the virus, due to the work of active search of infected family members.

### Inclusion criteria

Case Group: Individuals with HTLV-positive serology confirmed by the molecular biology method (PCR) for HTLV-1. Control Group: Individuals with HTLV-negative serology and PCR. Both groups: Individuals who agreed to participate in the study by signing the Free Informed Consent Term (FICT).

### Exclusion criteria

Individuals with inadequate samples for laboratory analysis. Individuals with HTLV-positive serology and not confirmed by molecular biology or confirmed for HTLV-2 or cases undetermined in serology and unconfirmed by molecular biology.

### Epidemiological research

Before the interview and the completion of a questionnaire with epidemiological data, the individuals were informed about the study and invited to participate in it, signing the Free and Informed Consent Form (FICF) in the event of an agreement. Blood was then collected for serological, molecular, and hematological examinations.

### Blood collection and sample processing

The blood collection was performed in 5 ml tubes containing the anticoagulant EDTA, previously identified with the names of the patients and their registration numbers corresponding to their care stipulated by the laboratory. The blood collection was obtained through venous puncture according to the safety recommendations of blood collection. After collection, the samples were sent to perform the blood count and later to perform other tests.

### Blood count

All blood samples collected from the patients were submitted to the blood count. These samples were previously homogenized and processed in the humacount30TS hematological automation equipment of 18 parameters with a three-part differential. The Hematological Counter is based on electrical and photocolorimetric impedance technology and consists of the following parameters: Erythrocyte count, hemoglobin dosage, hematocrit determination, mean corpuscular volume (MVC), mean corpuscular hemoglobin (HCM), mean corpuscular hemoglobin concentration (CHCM), and distribution amplitude of erythrocytes (RDW) that make up the erythrogram; leukocyte count, and leukocyte formula that the leukogram and platelet count. From all samples, two slides were made on smears and blushed for the differential counting of leukocytes and *the research of flower cells* and other atypical lymphocytes. The hematological parameters analyzed for the study were as follows: the count of leukocytes, lymphocytes, segmented, eosinophils, monocytes, hematocrit, red blood cells, hemoglobin concentration, and platelet count.

As a result of the different reference values cited in the literature for hematological parameters, in order to adapt all the variables in a single reference, reference values according to the literature were adopted in this study (Hoffbrand and Moss, 2013).

### Blood smear staining and differential leukocyte count

The blood smear was used to differentiate between leukocytes, that is, to make a count of the number of neutrophils, lymphocytes, monocytes, and eosinophils reaching a percentage of each cell found. The confection was made with a small drop of blood placed on a glass slide where a smear was made, dragging the drop of blood with another blade to form a film. The blood was homogenized before the smear was made so that the cells were well distributed. The smear was cordoned with Leishman dye, a yellowish eosin compound, and methylene blue oxidation products. Hence the differential count of leukocytes, with the count of 100 leukocytes in the blood smear, differentiating them according to their varieties of color and morphology observed from the microscope with the 100× lens.

### Flower cells and other atypical lymphocytes

After the preparation of a homogeneous and slender blood smear in a new, degreased, clean, and dry lamina, bleached by Leishman dye, the *flower cell and* other atypical lymphocytes were researched. The research was carried out through the blade throughout its length, using on average 50 fields with the immersion lens (40×), and when necessary for better visualization also used the 100× lens (Hoffbrand and Moss, 2013).

### International council for standardization in hematology recommendations for standardization of nomenclature and graduation of morphological changes in peripheral blood

Cell differentiation is a process that involves the identification of characteristics related to the size and shape of the nucleus, chromatin pattern, and size and aspect of the cytoplasm. In this context, the criteria for the classification of atypical lymphocytes follow:

• Abnormalities include increased cell size.
• Immaturity of the nucleus with the presence of nucleole and chromatin without condensation.
• Irregular nuclear contour or lobulation.
• Basophilia or cytoplasm vacuolization and irregular contour of the cell.
• The cytoplasm can be abundant with a color variation from pale blue to intense blue.

### The human T-lymphotropic virus type 1 enzyme immunoassay

For the identification of anti-HTLV-1/2 antibodies, qualitative and enzyme immunoassay, ELISA (Enzyme-Linked Immuno Sorbent Assay) was performed with the kit *Gold ELISA HTLV-1/2* (*REM - São Paulo, Brazil*) according to the manufacturer’s instructions at the Laboratory of Molecular and Cellular Biology of NMT/UFPA.

This test is used for the detection of IgM, IgG, and IgA anti-HTLV-1 and HTLV-2 antibodies in human serum or plasma by the ELISA method. It uses recombinant antigens (gp46 and gp21) fixed to the microplate. The positive samples and those with a value close to that of *cut-off* were analyzed in duplicate.

### DNA extraction

The blood samples from the individuals were submitted for DNA extraction. The method of extracting total DNA from mononucleated peripheral blood cells was used, according to the *Wizard Genomic DNA Purification* (*Promega Corporation, Madison, WI, USA*) *kit protocol*.

### Amplification of the human β-globin gene

All extracted genomic DNA was submitted to amplification of the human β-globin gene with primers G73 (5’-GAAGAGCCAAAGGACAGGTAC-3’) and G74 (5’-CAACTTCATCCCTCACC-3’) generating a fragment of 268 bp, whose objective is to evaluate DNA integrity and exclude the presence of PCR inhibitors.

In this procedure, 3.5 μL of GoTaq^®^ Green Master Mix *Purification* (*Promega Corporation, Madison, WI, USA*), 2.0 μL of autoclaved distilled water, 10 pmol/μL of each primer, and 1 μL of DNA were used for a final volume of 7 μL. The amplification protocol followed the denaturation temperature of 94°C for 5 min, followed by 30 cycles, 94°C for 45 s, 55°C for 45 s, and 72°C for 45 s, followed by the final extension temperature of 72°C for 10 min and 10°C for 10 min. The amplification of the segments mentioned above was performed in the thermocycler of *Biocycler MJ96* + */MJ96G* (*Applied Biosystems, Waltham, MA, USA*) (Tuke et al., 1992).

### Amplification and genotyping of the human T-lymphotropic virus type 1

Once the proviral DNA was amplified, a nested PCR was run, followed by enzymatic digestion for the confirmation of the HTLV infection and the differentiation of the virus types 1 and 2. For this, the pX region of the virus was amplified. This initial reaction was run in a solution containing 5.0 de *Go Taq Green Master Mix* (*Promega Corporation, Madison, WI, USA*), 2.0 μL of water, 1 μL (10 pmol) of each primer–HTLV_External F 5’-TTCCCAGGGTTTGGACGAAG-3’ (7,219–7,238, forward) and HTLV_External R 5’-GGGTAAGGACCTTGAGGGTC-3’ (7,483–7,464, reverse)–and 1.0 μL of the DNA, for a final volume of 10 μL.

In the second phase of the nested PCR, the same quantity of Go Taq Green Master Mix (Promega, Madison, WI, USA) was used, together with 1 μL (10 pmol) of the primers HTLV_internal F 5’CGGATACCCAGTCTACGTGTT3’ (7,248–7,268, forward) and HTLV_internal R 5’GAGCCGATAACGCGTCCATCG3’ (7,406–7,386, reverso), 2.5 μL of water and 0.5 μL of the product of the first PCR, producing a fragment of 159 bps.

Positive (a sample known to be infected) and negative controls were used for each PCR reaction. The products were electrophoresed (60 min at 100 V) in 2% agarose gel, stained with ethidium bromide in 1 × TAE buffer (TAE 50x stock—1.6 M Tris-Base, 0.8 M sodium acetate, and 40 mM EDTA-Na2 per liter of deionized water), and visualized using an ultraviolet transilluminator (Tuke et al., 1992).

### Enzymatic digestion (RFLP) to characterize human T-lymphotropic virus type 1

After the identification of cases with positive PCR results, using the 1% agarose gel, enzymatic digestion (RFLP—polymorphisms of the length of restriction fragments) of the amplified product was performed to identify the type of HTLV-1/2 present in the samples.

The RFLP reaction of this *pX* gene product (159 bp) was performed by mixing 6.0 μL of the amplified product, 7 μL of H20, 1.5 μL of buffer E (Promega, Madison WI, USA), and 0.5 μL of the *restriction enzyme Taq*I (*10 U/*μ*L, Promega, Madison WI, USA*), with subsequent incubation at 65°C for 2 h.

The presence of the restriction site (T/CGA) generates three fragments (21, 53, and 85 bp) in HTLV-2 and two fragments (21 and 138 bp) in HTLV-1.

The products of enzymatic digestion were visualized in agarose gel of 3% to 100 V for 45 min in 1 × TAE buffer (TAE 50 × stock-TrisBase 1.6 M, Na Acetate 0.8 M, and EDTA-Na240 Mm/1,000 ml of deionized water), using transilluminator in light with an ultra-violet light source (Tuke et al., 1992).

### Statistical analyses

All data from hematological parameters as well as all information collected in the interview and medical records were inserted into a spreadsheet using the Microsoft Office Excel© 2010 program.

The statistical tests were performed with the aid of the BioEstat version 5.0 program, to identify the frequencies and make the graphs and tables.

Initially, descriptive statistical analyses were performed, i.e., through graphs and frequency tables of the variables analyzed, estimation of mean, standard deviation, maximum, and minimum of numerical variables. The significance level to reject the null hypothesis was 5%, i.e., a *p*-value of 0.05< was considered statistically significant.

For the hematological parameters, the Z and Mann-Whitney tests were used to evaluate the relevant difference and evaluate the significance among the populations under study.

The Chi-square test (χ^2^) was used to compare the significant factors between the frequencies of patients and non-carriers of HTLV-1.

## Results

We investigated 202 individuals, including 87 positives for HTLV-1 (43.1%) and 115 individuals negative for HTLV-1 (56.9%), between September 2015 and August 2016.

Among the study samples, 72 individuals were male (35.6%), while 130 were female (64.4%). When the frequency of individuals negative for HTLV-1 infection was observed, it was found that 40.9% of these were male individuals, while 59.1% were female. When only individuals positive for HTLV-1 infection were analyzed, the frequency of male infected individuals was 28.7%, corresponding to 25 cases, while the female individuals corresponded to 71.3%, corresponding to 62 cases (Table 1), demonstrating a higher frequency of infection in individuals of this sex (*p* = 0.009).

**TABLE 1:** Distribution of the human T-lymphotropic virus type 1 (HTLV-1) infection regarding gender.

Gender	Total *n* (%)	Negative *n* (%)	Positive *n* (%)	*P*-value
Male	72 (35.6)	47 (40.9)	25 (28.7)	0.009
Female	130 (64.4)	68 (59.1)	62 (71.3)	

The mean age of HTLV-1-positive patients was 46.5 years with a standard deviation (SD) of 16.08 years varying ages between 17 and 81 years. In negative cases, the mean was 40.3 years, with a standard deviation of 17.60 ranging from 2 to 73 years of age.

**FIGURE 1:**
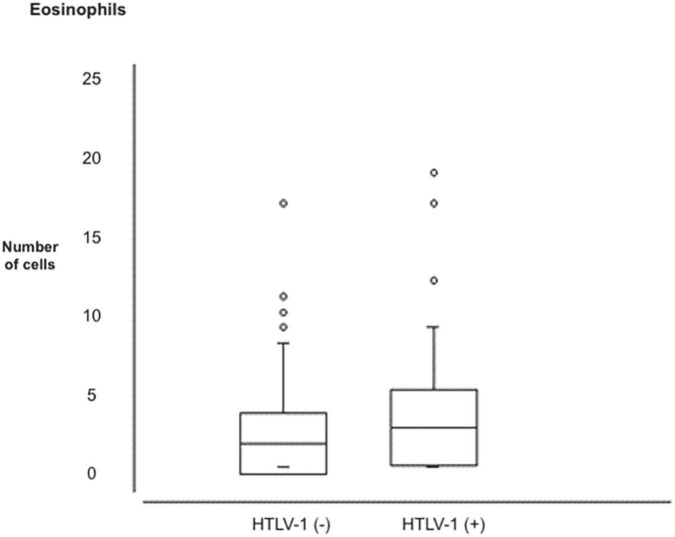
Distribution of eosinophils in patients negative and positive for the human T-lymphotropic virus type 1 (HTLV-1).

**FIGURE 2:**
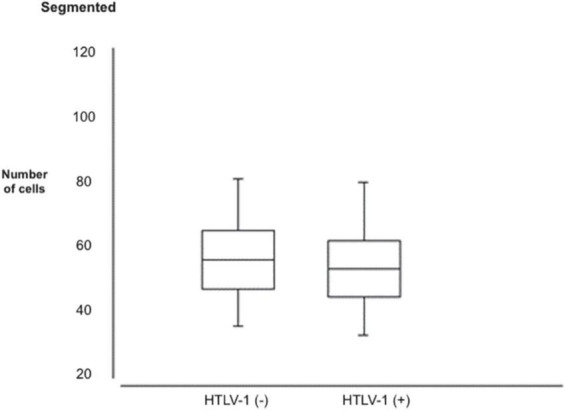
Segmented distribution in patients with negative and the human T-lymphotropic virus type 1 (HTLV-1) positive.

The minimum, maximum, mean, and standard deviation count is presented concerning age and hematological parameters (number of leukocytes, lymphocyte count, hemoglobin concentration, and other blood count parameters) in the study population (Table 2). In this table, the data presented in the eosinophil count (*p* = 0.0092) showed the increased eosinophilic ratio in HTLV-1 positive patients (Figure 1) and also a decrease in the presence of neutrophils in these HTLV-1-positive patients (*p* = 0.0303; Figure 2).

**TABLE 2:** Profile: Age and hematological parameter.

Negative (*n* = 115)					Positive (*n* = 87)				
Variable	Min.	Max.	Ave.	SD	Min.	Max.	Ave.	SD	*P*-value
Age	2	73	40.2	17.6	17	81	46.5	16.08	0.0090
Leukocyte	3,400	15,100	7,527	2,142	1,700	11,400	7,054	1,738	0.8290
Lymphocyte	9	56	34	9	10	58	35	9	0.3781
Segmented	35	84	57	10	32	83	54	9	0.0303
Eosinophilic	1	18	3	3	1	20	4	3.5	0.0092
Monocyte	3	8	6	1	3	10	6	1	0.7721
Red blood cell	3.00	6.10	4.65	0.5	1.85	5.82	4.50	0.6	0.1693
Hemoglobin	9.0	16.6	13.2	1.6	4.9	15.7	12.8	1.7	0.1490
Hematocrit	27.4	54.0	41.2	5.2	16.8	50.7	40.4	5.6	0.3127
Platelet (10^3^)	107	602	252	80.8	19	604	243	87.4	0.4686

SD, standard deviation; Min, minimum; Max, maximum; Ave, average.

The data show the presence of HTLV-1 positive individuals with a predominance of ages above 50 years. Table 3 shows the frequency distribution of positive and negative individuals between the ages mentioned; 20 years, between 20 and 30 years, 31 and 50 years, and over 50 years.

**TABLE 3:** Age distribution in the population studied by age group according to the presence of the human T-lymphotropic virus type 1 (HTLV-1) infection and gender.

Age	Negatives *n* (%) (*n* = 115)	Positives *n* (%) (*n* = 87)	Positives *n* (%) (Female)	Positives *n* (%) (Male)
<20	15 (13.1)	2 (2.3)	1 (2.6)	1 (4)
Between 20 and 30	23 (20.0)	15 (17.2)	13 (21.0)	2 (8)
Between 31 and 50	41 (35.6)	31 (35.6)	25 (40.3)	6 (24)
>50	36 (31.3)	39 (44.8)	23 (37.1)	16 (64)

Regarding the number of leukocytes, the data showed that 12 positive HTLV-1 patients (13.8%) presented a change in the number of leukocytes, taking into account the reference interval of 5,000–10,000/mm^3^ (Hoffbrand and Moss, 2013), being four patients with leukocytosis and eight patients with leukopenia (Table 4). HTLV-1 infection did not play a significant role in an increase in cases of individuals with altered leukocytes.

**TABLE 4:** Analysis of the number of leukocytes in cases with and without the human T-lymphotropic virus type 1 (HTLV-1) infection.

Negatives *n* (%)	Positives *n* (%)	*P*-value
Normal value 92 (80.0)	75 (86.2)	
Leukocytosis 14 (12.2)	4 (4.6)	0.1593
Leukopenia 9 (7.8)	8 (9.2)	

Table 5 shows the frequencies of patients positive and negative for HTLV-1, according to their lymphocyte counts. Most HTLV-1 positive individuals, as well as non-infected individuals, had normal lymphocyte count (*p* = 0.6191).

**TABLE 5:** Frequency analysis of lymphocyte count in the study population.

	Negatives *n* (%)	Positives *n* (%)	*P*-value
Lymphocyte > 45%	12 (10.5)	13 (14.9)	0.6191
Lymphocyte between 15 and 45%	101 (87.8)	72 (82.8)	
Lymphocyte < 15%	2 (1.7)	2 (2.3)	

**FIGURE 3:**
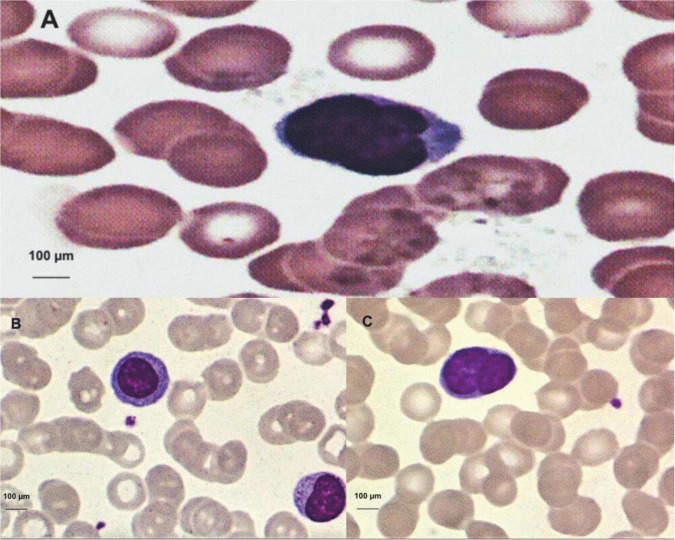
**(A)** Trilobulate atypical lymphocyte, image obtained through microscopic visualization, and the human T-lymphotropic virus type 1 (HTLV-1)-positive patient. **(B)** Atypical lymphocyte with large diameter and abundant cytoplasm (upper left). **(C)** Bilobulateaty atypical lymphocyte.

Atypical lymphocytes were identified only in the peripheral blood of HTLV-1 patients, in this case, in 20.7% (*n* = 18) and none of the negative cases presented lymphocytic atypical (*p* = 0.0001; Table 6). Figure 3 represent some atypical lymphocytes.

**TABLE 6:** Analysis of the frequency of the study population in relation to the absence or presence of atypical lymphocytes.

	Negatives *n* (%)	Positives *n* (%)	*P*-value
Absence of atypical lymphocytes	115 (100)	69 (79.3)	<0.0001
Presence of atypical lymphocytes	0	18 (20.7)	

It was found in the eosinophil count that eosinophilia above 15% was present at a frequency of 2.3% (*n* = 2) in HTLV-1 patients. Most of these patients had counts below 7% of eosinophils in their blood counts.

Regarding hemoglobin concentration, a percentage of 18.4% (*n* = 16) of patients below the cut-off reference (12–16 g/dL) and anemia was evidenced in these patients, and most individuals positive for HTLV-1 had a hemoglobin concentration above or equal to 12 g/dL.

Platelet count between cases with and without HTLV-1 infection was within the acceptable standard with a minimum reference of 150,000/mm^3^. Platelet count was not relevant concerning HTLV-1 infection, where six patients (6.9%) of those infected with HTLV-1 had thrombocytopenia, with a value below 150.000/mm^3^.

Among HTLV-1 positive patients (*n* = 87), 22 cases were studied with any sign, symptom, or clinical diagnosis related to infection (Table 7). Of these 22 studies, two had as diagnosis the myelopathy associated with HTLV-1/tropical spastic paraparesis (HAM/TSP), two presented furunculosis, and none presented any hematological alteration. One patient was identified with strongyloidiasis with alteration in his profile in the red blood series, with red blood cells, hematocrit, and altered hemoglobins. Another positive individual had fatigue associated with anemia with a hemoglobin concentration below 7.9 g/dL.

**TABLE 7:** Clinical findings of the human T-lymphotropic virus type 1 (HTLV-1)-positive patients associated with hematological changes.

	HAM/TSP *n* (%)	Strongilodiasis *n* (%)	Furunculosis *n* (%)	Anemia/Fatigue *n* (%)
HTLV-1 (+)	2 (9.1)	1 (4.5)	2 (9.1)	1 (4.5)
Hematological change	S/A	RBC: 2.81 Hb: 4.9 Ht: 1.0.8	S/A	Hb < 7.9

S/A, no hematological alteration; RBC, red blood cell; Hb, hemoglobin; Ht, hematocrit.

## Discussion

These data presented in the study of the hematological profile of HTLV-1-infected individuals in patients treated at the Tropical Medicine Center in the metropolitan region of Belém do Pará contribute to the knowledge about the distribution and presentation of the characteristic of this population concerning hematological parameters. The data presented here through this study can show a hematological profile of HTLV-1 positive patients and show alterations that may help in an investigation of a possible trigger of an aggravating disease of this viral disease, as well as may enable better follow-up and counseling of infected patients.

The blood count of HTLV-1 individuals represents a collection of tests, which analyze quantitatively and qualitatively the morphology of the blood figurative elements. Therefore, the knowledge of hematological characteristics for the population studied in this experiment becomes fundamental, since there are few hematological reference patterns in this population.

Regarding the sociodemographic profile, there was a higher frequency of individuals positive for HTLV-1 female (71.3%), aged between 31 and 50 years (40.3%), characteristics similar to those found in a study conducted in Belém on the clinical-epidemiological profile of HTLV-1 infected patients (Glória et al., 2015), who showed the predominance of females for HTLV-1. The predominant age group in the study between males and females for HTLV-1 patients was above 50 (44.8%).

The existence of a higher tendency of infection in females may suggest that they are more susceptible to HTLV infection, due to a possible more efficient sexual transmission from man to woman, possibly associated with a greater amount of infected and viable lymphocytes released during male ejaculation as described by other authors (Catalan-Soares et al., 2004; Moriuchi and Moriuchi, 2004). In childhood, HTLV-1 seropositivity is very low and increases from adolescence to early adulthood. In women, this increase is more pronounced and continues after 40 years, while in men the positivity is lower and reaches a plateau after 40 years (Romanelli et al., 2010).

The results of the blood count parameters found in this study were presented in tables, showing the point values for each blood count parameter referring to the positive and negative population for HTLV-1 as well as its minimum, maximum, mean, and standard deviation values (Table 2). Through the z-test for independent samples, the table indicates two parameters with abnormalities in their distribution in the concentrations among the studied population; segmented ones and eosinophils.

The increase in eosinophils may be related to the presence of co-infection, especially Strongyloides or another parasitosis, or the process of leukemogenesis itself. Plumelle et al. (1993) suggested that hypereosinophilia affects the prognosis of HTLV-1-associated leukemia. Persistent eosinophilia with no definite cause may be an indicator of the progression of malignant neoplastic disease, ATL (Ohshima et al., 1998). This profile of the HTLV-1-infected individual has an increased eosinophilic mean as its immunologic response compared to HTLV-1-negative individuals.

Studies suggest that HTLV-1 infection is associated with immunosuppression, HTLV-1 is a slow-progression virus with low virulence (Proietti et al., 2005). Thus making sure that there is no significant increase in leukocytes in these infected patients. The joint analysis of leukocyte data, without differentiating by gender and age group, allows an overview of this hematological profile of the population. However, due to the normal variation, we present in the leukocyte count, it was generally observed that individuals with HTLV-1 infection did not cause a significant differentiation in the total leukocyte count, either below the stipulated reference (reference value 5,000–10,000/mm^3^) or above this reference.

When we have a viral process, it is common for the number of lymphocytes to increase. However, HTLV-1 responds to infection preferably peripheral T cells, predominantly CD4+ T lymphocytes (Xie and Green, 2005) and acting latently in most T cells, this host immune response to viral infection is a crucial event, determining the direction of the immune response and a profile of lymphocyte response influencing its hematological count. Among the lymphocyte count for HTLV-1 positives, only 13 in the total population of 87 had lymphocytosis (14.9%), i.e., lymphocytes above 45%. The comparison of lymphocytes and leukocytes through their count did not show a relationship to confirm statistically direct between the increase of leukocytes at the expense of lymphocytes, not showing a significant variation in the overall leukocyte count.

Lymphocytosis accompanied by leukocytosis requires greater attention and may have the presence of atypical lymphocytes and may correspond to more than 10% of circulating lymphocytes. Atypical lymphocytes are highly pleomorphic, many of them large with a diameter between 15 and 30 μm, with abundant cytoplasm, and intensely basophilic. Some have large central nucleolus, nuclei can be round, oval, reniform, lobulated, or occasionally clover-shaped. Lymphocyte morphology may be the first sign of the diagnosis of ATL (Shuh and Beilke, 2005). These cells may appear with significant nuclear abnormalities such as the multilobulate nucleus *called flower cells*. Considering that ATL presents distinct clinical forms, Shimoyama (1991) classified it into four types, based on disease extension, evolution time, lymphocytic and biochemical changes. The acute form presents abnormal circulating lymphocytes and *flower* cells, with infiltration of lymph nodes and viscera, as well as tumor manifestations. Symptoms are usually non-specific and may appear in any lymphoproliferative disease.

Chronic and latent forms have a better prognosis, but may evolve to the acute form at any time and have no lymphocytosis. The HTLV-1 genome is randomly integrated into the cellular genetic material. In HTLV-1 infection there is little or no vírion in the plasma of the infected individual, reflecting the main viral replication pathway, viral synapse (Plumelle et al., 1993; Ohshima et al., 1998; Shuh and Beilke, 2005; Romanelli et al., 2010).

Studies show that HTLV-1-infected CD4+ T lymphocytes persist for about 7 years in the same individual, suggesting that the virus participates in the process of transformation and immortalization of these lymphocytes. After HTLV-1 infection, the latency period is long (about 50 years), indicating that in the development of ATL multiple steps are required, accumulating genetic mutations in infected cells (Plumelle et al., 1993; Ohshima et al., 1998; Shuh and Beilke, 2005; Romanelli et al., 2010).

The probability of an HTLV-1 carrier developing ATL in the course of prolonged latency shows that the process of cell transformation and leukemogenesis should involve many complex events within the host’s T-cells (Shuh and Beilke, 2005).

Lymphocytes with different morphology, which can be found in the peripheral blood of healthy patients in a small percentage (1–4%) called atypical lymphocytes, were also found in HTLV-1-positive patients where 18 of them (20.7%) had at least one of these lymphocytes, and in two patients had a count above the reference range, one with 5% of atypical lymphocytes and the other with 7% of atypical lymphocytes, the negative population did not even present a count of these lymphocytes. No lymphocyte with the flower-shaped nucleus of positive patients was found.

About eosinophils, 9.5% (*n* = 11) of negative patients had eosinophilia between 7 and 14%, and among the positive 14.9% (*n* = 13) had eosinophilia between 7 and 14%, alteration sofa not related to either an allergic process or any parasitosis, and there was no difference in frequency between populations.

As for hemoglobin concentration, specifically, the data showed that, of the 87 HTLV-1-positive patients, 12 (13.8%) had anemia, with hemoglobin ranging from 10.0 to 11.9 g/dL, taking as a cutoff point 12.0 g/dL, especially 4 HTLV-1 patients (4.6%) presenting hemoglobin below 10 g/dL and generally a normal distribution among the population (*p*-value = 0.6245).

Also, evaluating platelet count in the population, with a cut-off value of 150,000 platelets/mm^3^, we obtained a 5.2% (*n* = 6) below this profile for negative patients and 4.6% (*n* = 6) among the eight patients with lower-normal platelet concentration positive for HTLV-1, and two of them with a score below 100,000/mm^3^, patients without having problems with heavy bleeding. There was normality regarding the distribution of platelet concentrations among the population studied.

The human T-lymphotropic virus type 1 infection can lead to numerous changes from the immunological, oncological-hematological, neurological, rheumatological, infectious, and ophthalmological points of view. HTLV causes persistent infections throughout life and this virus is associated with serious diseases such as ATL. Most infected individuals remain asymptomatic throughout their lives, and about 5% develop these diseases (Poetker et al., 2011). HTLV-1 is a retrovirus that functionally alters important cells of the immune system. Unlike other viral infections, individuals infected with HTLV-1 do not receive antiretroviral treatment due to the inefficiency of these drugs against HTLV-1; therefore, HTLV-1 treatment is restricted to associated diseases.

Analyzing the hematological parameters, a correlation was found between the positivity of the virus and the presence of some symptoms and clinical diagnoses determined in these positive patients, showing that two patients with a confirmed diagnosis of HAM/TSP according to clinical evaluation criteria performed in these patients (Costa, 1996) who did not present changes in hematological parameters in their blood counts. One of the patients among the positively presented strongyloidiasis, an intestinal parasitosis that causes a decrease of iron in the body causing its deficiency and causing an iron deficiency anemia. This patient had a significant alteration in the red blood cell count (2.81 106/mm^3^), hemoglobin (4.9 g/dL), and hematocrit (16.8%) values below the reference (Hoffbrand and Moss, 2013). In one of these patients, furunculosis was observed without hematological alteration, and three presented anemia with fatigue symptoms identified with hemoglobin below 7.9 g/dL, which explains this symptom presented since a reduction in hemoglobin in the blood can cause symptoms such as fatigue.

## Conclusion

It was detected that the highest frequency of HTLV-1 was among women, affecting mainly those older than 50 years. Among the hematological parameters, the eosinophilic and neutrophilic profiles of HTLV-1-infected patients stood out, with a relevant association with infection.

There was no significant change in the total number of leukocytes and lymphocytes in HTLV-1 infected patients. The presence of atypical lymphocytes in HTLV-1 infected lymphocytes was significantly higher than in non-infected lymphocytes and these abnormalities in lymphocytes should always be monitored through the blood count.

The human T-lymphotropic virus type 1-infected patients present symptomatic changes to different degrees and should always be evaluated and monitored through tests such as the blood count. The results of this study indicate the need for further studies of hematological parameters for a more comprehensive description of cases of HTLV-1 infection.

## Data availability statement

The original contributions presented in this study are included in the article/supplementary material, further inquiries can be directed to the corresponding author.

## Ethics statement

This present study was submitted and approved (CAAE: 31014114.2.0000.5172) by the research ethics committee of the NMT/UFPA, in compliance with the norms and guidelines of the Federal University of Pará. The patients/participants provided their written informed consent to participate in this study.

## Author contributions

All authors listed have made a substantial, direct, and intellectual contribution to the work, and approved it for publication.
